# CMV2b-Dependent Regulation of Host Defense Pathways in the Context of Viral Infection

**DOI:** 10.3390/v10110618

**Published:** 2018-11-09

**Authors:** Jian-Hua Zhao, Xiao-Lan Liu, Yuan-Yuan Fang, Rong-Xiang Fang, Hui-Shan Guo

**Affiliations:** 1 State Key Laboratory of Plant Genomics, Institute of Microbiology, Chinese Academy of Sciences, Beijing 100101, China; lyueyawanwan@163.com (X.-L.L.); fangyy@im.ac.cn (Y.-Y.F.); fangrx@im.ac.cn (R.-X.F.); 2 College of Life Science, University of the Chinese Academy of Sciences, Beijing 100101, China

**Keywords:** CMV, RNAi, VSRs, 2b protein, SA

## Abstract

RNA silencing (or RNA interference, RNAi) plays direct roles in plant host defenses against viruses. Viruses encode suppressors of RNAi (VSRs) to counteract host antiviral defenses. The generation of transgenic plants expressing VSRs facilitates the understanding of the mechanisms of VSR-mediated interference with the endogenous silencing pathway. However, studying VSRs independent of other viral components simplifies the complex roles of VSRs during natural viral infection. While suppression of transgene silencing by the VSR 2b protein encoded by cucumber mosaic virus (CMV) requires 2b-small RNA (sRNA) binding activity, suppression of host antiviral defenses requires the binding activity of both sRNAs and AGOs proteins. This study, aimed to understand the functions of 2b in the context of CMV infection; thus, we performed genome-wide analyses of differential DNA methylation regions among wild-type CMV-infected, CMVΔ2b-infected, and 2b-transgenic *Arabidopsis* plants. These analyses, together with transcriptome sequencing and RT-qPCR analyses, show that while the majority of induced genes in 2b-transgenic plants were involved in extensive metabolic pathways, CMV-infection 2b-dependent induced genes were enriched in plant immunity pathways, including salicylic acid (SA) signaling. Together with infection with CMV mutants that expressed the 2b functional domains of sRNA or AGO binding, our data demonstrate that CMV-accelerated SA signaling depends on 2b-sRNA binding activity which is also responsible for virulence.

## 1. Introduction

RNA silencing (or RNA interference, RNAi) is an evolutionarily conserved and inducible defense pathway that specifically targets and inactivates invading nucleic acids [1,2,3,4]. Small RNAs (sRNAs) are the key mediators of RNAi and are mainly classified into two major types, microRNAs (miRNAs) and small interfering RNAs (siRNAs), which regulate gene expression at the post-transcriptional level (PTGS) or transcriptional level (TGS) in association with RNA-directed DNA methylation (RdDM), respectively [1,5]. RNAi-mediated antiviral plant immunity is triggered by double-stranded RNA (dsRNA) derived from the replication intermediates of RNA viruses, bidirectional transcription of DNA viruses and imperfectly base-paired intermolecular hairpins in viral genomes or transcripts [2,3,4,6,7]. During virus infection, these dsRNAs are processed by the RNase III-type enzyme Dicer-like (DCL) protein into sRNAs, which are virus-derived siRNAs (viRNAs). viRNAs are incorporated into the plant Argonaute (AGO) protein, forming an RNA-induced silencing complex (RISC) to cleave the viral RNA or suppress the transcription of DNA viruses via the RdDM pathway [2,7,8,9,10]. These viRNAs, as primers for host RNA-dependent RNA polymerase (RDR), are expected to initiate the production of dsRNAs from viral RNA templates and generate secondary viRNAs [3,11,12]. Beyond the limitation of viral RNA accumulation in initially infected cells by processing viral dsRNAs into viRNAs, RNAi-mediated antiviral activity contributes to achieving a robust systemic defense response [2,3]. For instance, viRNAs move short and long distances, giving distant tissues the ability to target the incoming virus before it replicates, and blocks the viral infection [13].

In addition to directly targeting viral RNAs, growing evidence indicates that RNAi has been implicated in resistance (R) genes basal immunity and hormone signaling [13]; R gene products (R proteins) are thought to recognize viral coat, movement or replication proteins [14] and to initiate rapid changes in host cell physiology and metabolism to against viruses [15]. A coevolutionary model of plant R genes and miRNAs has been illustrated by analyzing diverse land plants [16]. To minimize the cost and avoid autoimmunity reactions, R genes are silenced by TGS or PTGS in the absence of pathogens [8,17]. For example, the R gene *N* undergoes regulation by nat-miR6019 and nat-miR6020 in tobacco [18]. Phytohormones, such as salicylic acid (SA), jasmonate (JA), ethylene (ET), abscisic acid (ABA), auxin, gibberellins (GAs), cytokinins (CKs) and brassinosteroids (BRs), are also important regulators of plant defenses [19], and SA and JA are major defense-related phytohormones [19,20].

RNAi plays a major role in maintaining the balance between investment in growth and defenses against infection by pathogens, including viruses [4]. To facilitate infection, viruses independently encode suppressors of RNAi (VSRs), which act at different steps of RNAi through diverse mechanisms to counteract antiviral defenses [2,4,8,13]. During viral infection, VSRs can hamper sRNA synthesis and stability, inhibit AGO and DCL function, degrade AGO1 through autophagy or 26S proteasome-mediated degradation, or impede systemic transmission of RNAi signals [8,13]. Some VSRs can be recognized by R protein, and then elicit a hypersensitive response (HR) as the effectors of nonviral pathogen triggered immunity [4,21]. VSRs also induce host defense gene expression by suppressing PTGS- and TGS-mediated repression [22,23,24]. The majority of R proteins comprise nucleotide-binding and leucine-rich repeat domains (NBS-LRRs) [25]. In virus-infected tomato, miR482- and RDR6-dependent secondary siRNAs modulated NBS-LRRs to enhance host immunity [13,24]. The multiple functions of VSRs have been decrypted by studying them independent of other viral components. However, the innate role of VSRs in the context of virus infection is more requisite to elaborate on the interaction between host and viruses. Indeed, the heterologous expression of VSRs did not always functionally complement VSR mutant viruses [26], suggesting that transgene-derived constitutive expression of VSRs might have different actions/effects that virus expression during natural viral infection.

The 2b protein encoded by the cucumber mosaic virus (CMV) acts at multiple processes, to suppress PTGS and TGS [27,28,29,30]. In previous studies, we identified two nuclear localization signals (NLSs), a dsRNA-binding domain and an AGO-interacting domain, in the 2b protein [30]. In 2b-transgenic plants, we found that 2b suppression of RdDM correlated with its binding to diverse length sRNAs and induced multicopy transposable elements (TEs) reactivation by reducing methylation [31]. The suppression of PTGS and DNA methylation by 2b required siRNA-binding activity independent of its ability to interact directly with AGOs [30]. Furthermore, we found that the 2b-AGO interaction redistributed the localization of both the 2b and AGO proteins in the nucleus, inhibited AGO1 slicer activity in vivo, and suppressed RDR-dependent antiviral silencing in the context of CMV infection [29,30,32]. To suppress excess virus accumulation, 2b has been inferred to be involved in inducing host antiviral defenses. 2b-mediated suppression of RdDM in host repetitive sequences might be involved in the biosynthesis of CMV satellite RNA (satRNA), which produced anti-CMV siRNAs and alleviated viral symptoms [6,10,33,34]. 2b might also be responsible for the induction of SA-mediated resistance to CMV in SA pretreated plants [35,36]. It was recently reported that the CMV CP protein negatively regulates the accumulation of 2b protein during late infection, leading to reduced CMV RNAs and postponing symptom appearance in newly emerging leaves [37]. Moreover, a recent study uncovered a molecular mechanism in which 2b, instead of using its VSR activity, directly interacted with JAZ proteins, which are the key repressors of JA signaling, and prevented JAZ degradation to inhibit JA signaling [38]. Taken together, these data reveal that the CMV2b protein serves as a central hub to balance virus accumulation and plant growth during CMV infection.

The present study aimed to uncover the effects of 2b protein in the context of virus infection; thus, we compared the differential DNA methylation regions (DMRs) in CMV-infected, and 2b-deficient CMV (CMVΔ2b)-infected, and 2b-transgenic plants, in which the DNA methylation levels varied significantly compared to Col-0 plants. This comparison together with transcriptome sequencing analysis, showed that 2b-dependent changes in DNA methylation was occurred on a larger scale in 2b-transgenic plants than in CMV-infected plants. Remarkably, while the majority of induced genes in 2b-transgenic plants were involved in extensive metabolism pathways, CMV-infection 2b-dependent-induced genes were specifically enriched in plant immunity pathways, including accelerating salicylic acid signaling.

## 2. Materials and Methods

### 2.1. Plant Growth and Virus Inoculation

Wild-type and 2b-transgenic (the 2b-3 line) *Arabidopsis* Col-0 seeds were imbibed on an Murashige and Skoog (MS) medium for 2 days at 4 °C. Seed were moved to a greenhouse with 16 h light and 8 h dark at a constant temperature of 23 °C, and after 10 days they were transplanted. Western blot was used to confirm 2b protein was stably expressed in 2b-3 plants (Figure S1).

In this study, infectious clones of CMV and CMV mutants (including CMVΔ2b, CMV2b_(1–76)_, and CMV2b_(18–111)_ that are with 2b-deficient or with the 2b functional domains of sRNA or AGO binding) that were described previously [32,34] were used to infect three-week old wild-type Col-0 plants. Inoculations were carried out using sap from systemically infected tobacco leaves. Sap was prepared by grinding leaves in a 5 mM sodium phosphate buffer (pH 7.2). Clarified sap was applied to carborundum-dusted leaves by mechanical rubbing. Buffer-treated plants were used as a negative control. Plants were covered for 24 h and then transferred to the greenhouse. For DNA isolation for Bisulfite sequencing, at 9 days post-inoculation (dpi), leaves from CMV-infected and CMVΔ2b-infected plants and mock-inoculated plants, as well as 2b-3 transgenic plants were collected, each from 32 plants, and pooled. For RNA assays, at 9 and 21 dpi, leaves were collected, each from 32 plants, and pooled, and then RNA isolation was used. Experiments were repeated three times.

### 2.2. DNA Bisulfite Sequencing and Bioinformatics Analyses

Genomic DNA was extracted with the CTAB method [39]. DNA sodium bisulfite treatment, library construction, and bisulfite sequencing were accomplished by BGI (http://www.genomics.cn/en/index). Clean reads were aligned to the *Arabidopsis* reference genome (TAIR 10, https://www.arabidopsis.org/). Four sets of DNA methylation data were obtained, including CMV-infected and CMVΔ2b-infected plants and mock-inoculated plants, as well as 2b-3 transgenic plants (referred to as CMV-plants, CMVΔ2b-plants, Col-0, and 2b-3 plants). BGI performed the basic analysis and DMR identification, as described in our previous study [31]. By comparing data between CMV-plants and Col-0, CMVΔ2b-plants and Col-0, 2b-3 plants and Col-0, three datasets of DMRs were obtained for analysis in this study. The overlapping DMRs were searched with a Perl script (https://www.perl.org/). The boxplot (Figure 2A) and heatmap (Figure 2B) of the methylation levels of DMRs were generated using R scripts (https://www.r-project.org/).

### 2.3. Transcriptome Sequencing

Total RNA was isolated with TRIzol reagent (Invitrogen, Carlsbad, CA, USA) according to the instruction manual. Library construction and transcriptome sequencing were accomplished by BGI. BGI performed the basic analysis and differentially expressed gene identification as description in our previous study [31]. Reads per kb transcriptome per million reads (RPKM) was used to calculate gene expression [40]. The false discovery rate (FDR) was used to correct the diversity test *P*-value [41]. Compared to Col-0, genes with *P* < 0.05 and FDR < 0.001 were included in our analysis.

AgriGO [42] was used to detect the enriched gene ontology (GO) [43] terms of differentially expressed genes with singular enrichment analysis (SEA). Enrichment GO terms will be found out after Fisher test (*P* < 0.05) from pre-calculated background or customized one [42]. Cross comparison of SEA was completed by SEACOMPARE [42].

The overlapping induced or suppressed genes were analyzed using the Draw Venn Diagram tool (http://bioinformatics.psb.ugent.be/webtools/Venn/).

### 2.4. Reverse Transcription Quantitative Real-Time PCR

For reverse transcription quantitative real-time PCR (RT-qPCR), total RNA was digested genomic DNA using DNase I (TaKaRa, Kyoto, Japan), then reverse transcribed into cDNA using M-MLV reverse transcriptase (TaKaRa, Kyoto, Japan). cDNA was subjected to RT-qPCR with a 1000 series Thermal Cycling Platform (Bio-Rad, Hercules, CA, USA) using EvaGreen 2×qPCR Mastermix (Applied Biological Materials Inc., Richmond, BC, Canada). *AT4G33380* was included in the assay for normalization [44]. At least three biological replicates within an experiment for each sample were performed. The 2^−∆∆CT^ method was used for relative quantification of each amplified product. Primer sequences are listed in Table S1.

### 2.5. RNA Gel Blotting Analysis

For the detection of viral RNAs, 15 µg of total RNA was separated on a 1.2% agarose denaturing gel containing 0.1% formaldehyde. The mixture of three 1-kb fragments at the 3′-terminus of each CMV cDNA clone were amplified and labeled with [α-^32^P] dCTP was used as probe. A total of 30 µg RNA was separated on 17% polyacrylamide-8 M urea gels for viral sRNA detection. The mixture of DNA oligonucleotides [32] specific to CMV RNA3 labeled with [r-^32^P] using T4 PNK (NEB, M0201V, Ipswich, MA, USA) was used as probe. RNA blotting signals were detected using ImageQuant TL 7.0 software (GE Healthcare, Cincinnati, OH, USA).

## 3. Results

### 3.1. Comparison of The Differential DNA Methylation Regions Between CMV-Infected and 2b-Transgenic Plants

**Figure 1:**
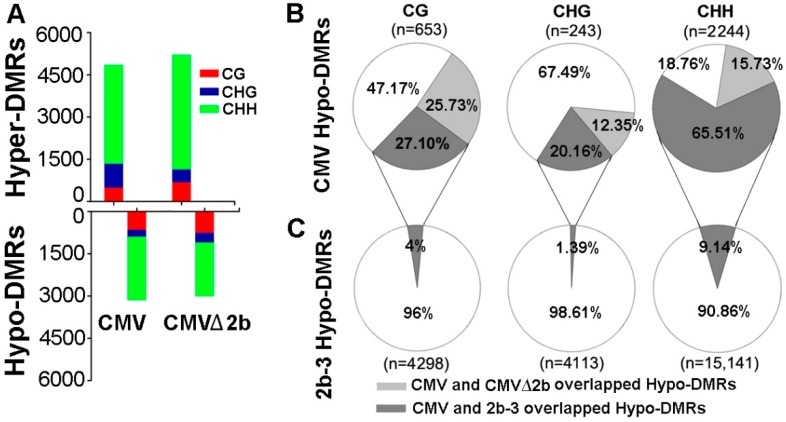
Comparison of the DMRs identified in CMV-, CMVΔ2b- and 2b-3 plants compared to Col-0 plants. (**A**) Comparison of the methylation levels of Hyper- and Hypo-DMRs in CG, CHG and CHH contexts in CMV-, CMVΔ2b- plants compared to Col-0 plants. (**B**) The Hypo-DMRs identified in CMV-plants overlap with those identified in CMVΔ2b- and 2b-3 plants. (**C**) The overlap of Hypo-DMR identified in 2b-3 plants with that identified in CMV-plants. The numbers of Hypo-DMRs loci in each context are shown (*n*).

**Figure 2:**
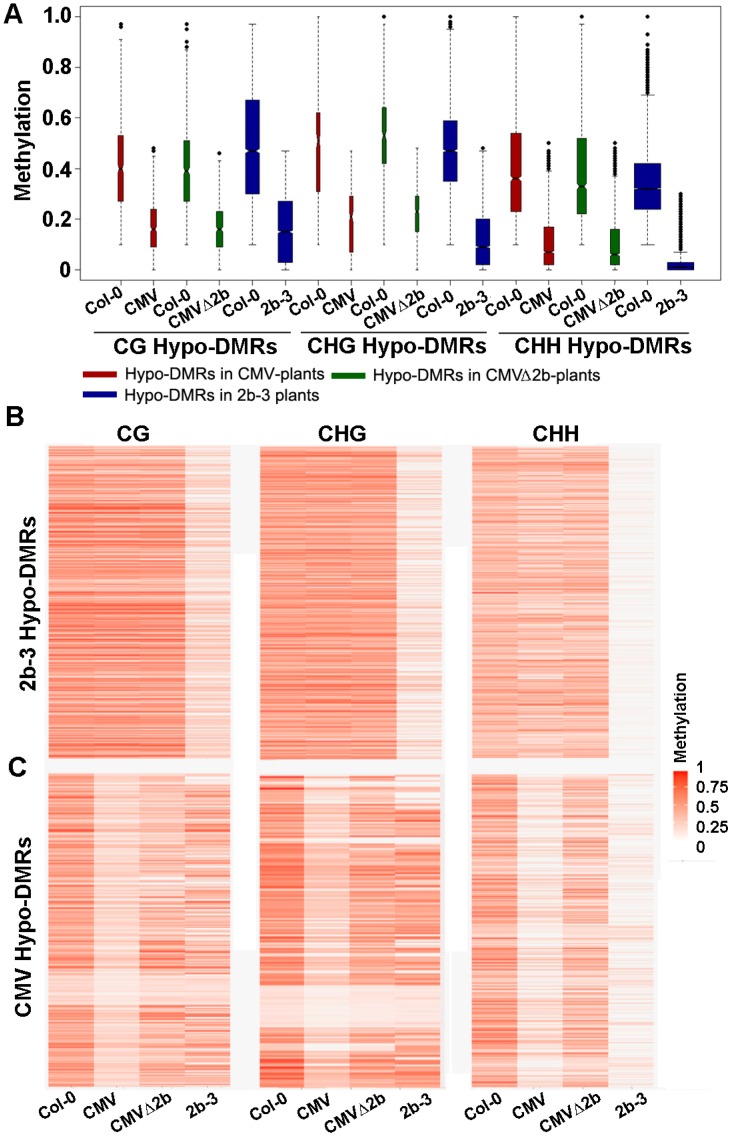
The methylation level of Hypo-DMRs in CMV-, CMVΔ2b- and 2b-3 plants. (**A**) Boxplots of the methylation level of each Hypo-DMR in CG, CHG, and CHH contexts in CMV-, CMVΔ2b- and 2b-3 plants. The width of the column indicates the quantity of Hypo-DMRs. (**B**,**C**) Heat map of the methylation level within each Hypo-DMR identified in 2b-3 plants (**B**) and CMV-plants (**C**). The degree of methylation to each Hypo-DMR coincided with the degree in red for each heat map.

To identify the function of 2b protein in inhibiting host DNA methylation during CMV infection, we first performed bisulfite sequencing on CMV- and CMV∆2b-infected plants (simplified as CMV-plants and CMV∆2b-plants) and identified the DMRs compared with wild-type Col-0 plants as previously described [31]. This approach led to the identification of 3140 hypomethylated DMRs (Hypo-DMRs) and 4848 hypermethylated DMRs (Hyper-DMRs) in CMV-plants (Figure 1A). Notably, this result was different from that for the 2b-3 line of 2b-transgenic plants, in which the DMRs were dominated by 23,552 Hypo-DMRs (and 5093 Hyper-DMRs) when compared with wild-type Col-0 plants (*P* < 0.001, chi-square test) [31]. The quantity of Hypo-DMRs in CMV-plants was also much less than that in 2b-3 plants (Figure 1B,C and Figure 2A). In CMV∆2b-plants, we also identified much more Hyper-DMRs (5207) than Hypo-DMRs (3002) (Figure 1A). We previously found that stable expression of the 2b protein mainly reduced genome-wide CHH and CHG (where H = A/T/C) methylation in 2b-3 plants [31]; thus, our analysis focused on the Hypo-DMRs.

We separated these Hypo-DMRs into each cytosine context. In agreement with the observation of 2b undermining the RdDM pathway through binding sRNAs [30,31], a high proportion (2244/3140) of CHH Hypo-DMRs was found in CMV-plants (Figure 1A). However, the same trend was also observed in CMV∆2b-plants, in which a proportion of CHH Hypo-DMRs was also obtained (Figure 1A). This finding led us to analyze the correlation among the Hypo-DMRs identified from CMV-, CMV∆2b- and 2b-3 plants. In CMV-plants, only 25.73%, 12.35% and 15.73% of CG, CHG and CHH Hypo-DMRs, respectively, overlapped with those in CMV∆2b-plants (Figure 1B), suggesting that reduced methylation of the Hypo-DMRs in CMV-plants largely required 2b activity. Indeed, while 27.11% of CG Hypo-DMRs and 20.16% of CHG Hypo-DMRs overlapped with those in 2b-3 plants (Figure 1B), up to 65.51% of CHH Hypo-DMRs overlapped with those in 2b-3 plants, demonstrating that reduced methylation of these loci, especially, in the CHH context, in CMV-plants was associated with 2b VSR activity during CMV infection (Figure 1B). However, these Hypo-DMRs overlapping with CMV-plants were only a small proportion of the Hypo-DMRs identified in 2b-3 plants (Figure 1C), and a large number of Hypo-DMRs identified in 2b-3 plants were not found in CMV-plants. This finding indicates that the effect of a transgene-expressed VSR, such as 2b, might not always reflect the effect of the VSR in the context of viral infection.

Next, we investigated the methylation level of these Hypo-DMRs. We previously found that 2b targeted endogenous DNA regions with a high density of methylation [31]. Similarly, we found that high methylation levels at loci in wild-type Col-0 corresponded to those Hypo-DMRs, from either CMV- or CMV∆2b-plants (Figure 2A). Distinctly lower CHG and CHH methylation in these Hypo-DMRs in 2b-3 plants (*P* < 0.05, Mann–Whitney test) was noted compared to that in CMV- and CMV∆2b-plants (Figure 2A), revealing strong impairment of CHG and CHH methylation with stable expression of the 2b protein. Figure 2B clearly shows high methylation levels in Col-0 at loci corresponding to Hypo-DMRs in 2b-3 plants (*P* < 0.05, *t* test). The level of CHH methylation at most loci was slightly reduced in CMV-plants (Figure 2B), in agreement with the finding that only 9.14% of CHH Hypo-DMRs in 2b-3 plants were found in CMV-plants (Figure 1C). Moreover, consistent with small proportions of CG, CHG and CHH Hypo-DMRs from CMV-plants overlapping with those in CMV∆2b-plants (Figure 1B), reduced methylation of CG, CHG, and CHH contexts at these loci was greater in CMV-plants than in CMV∆2b-plants (*P* < 0.05, *t* test) (Figure 2C). However, while reduced CG and CHG methylation at these loci was less in 2b-3 plants than in CMV-plants (*P* < 0.05, *t* test) (Figure 2C), comparatively reduced CHH methylation at these loci were observed in both CMV- and 2b-3 plants (Figure 2C), consistent with the high proportion (65.51%) of CHH Hypo-DMRs in CMV-plants overlapping with that in 2b-3 plants (Figure 1B). These data further demonstrate that CMV impaired CHH methylation mainly depended on the function of 2b during virus infection and suggest that CMV infection also caused 2b-independent demethylation of endogenous genomic sequences. Our data also show that 2b targeted extensive sites and had a stronger effect on inhibiting DNA methylation when 2b was expressed as a transgene in plants.

### 3.2. 2b Protein Differently Modulated Gene Expression in CMV-Infected and 2b-Transgenic Plants

**Figure 3:**
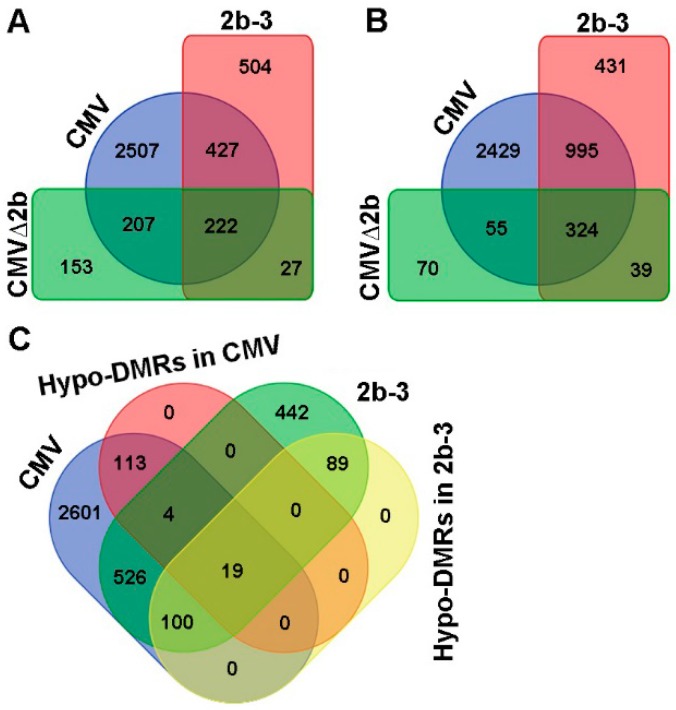
Compared to Col-0, the differentially expressed genes in CMV-, CMVΔ2b- and 2b-3 plants. (**A**) The induced genes were detected concurrently in CMV-, CMVΔ2b- and 2b-3 plants. (**B**) The suppressed genes were detected concurrently in CMV-, CMVΔ2b- and 2b-3 plants. (**C**) The induced genes correlated with Hypo-DMRs in CMV- and 2b-3 plants.

Next, we compared the transcription levels of genes in Col-0, CMV-, CMV∆2b- and 2b-3 plants by transcriptome sequencing. Compared to Col-0 plants, we found that 3363 genes were induced and 3803 genes were suppressed by CMV infection (*P* < 0.05, FDR < 0.001, Fold > 2) (Figure 3A,B). In the absence of the 2b protein, CMV∆2b infection only induced 609 genes and suppressed 488 genes; among these genes, 70.4% (429/609) and 77.7% (379/488) of induced and suppressed genes, respectively, were concurrently detected in CMV-plants (*P* < 0.05, FDR < 0.001, Fold > 2) (Figure 3A,B). These data demonstrate that the expression of most CMV-altered genes requires the function of 2b. During CMV infection, 2507 of the 3363 induced genes (74.5%) and 2429 of the 3803 (63.9%) suppressed genes occurred in the presence of the 2b protein, suggesting that the gene expression changes required the VSR activity of 2b and presumably cooperation between 2b and other viral components. Indeed, in the absence of viral infection, in 2b-3 plants, only 1180 induced and 1789 suppressed genes were detected compared to those in Col-0 plants (*P* < 0.05, FDR < 0.001, Fold > 2) (Figure 3A,B). Among these genes, 55% (649/1180) of induced genes and 73.7% (1319/1789) of suppressed genes were concurrently detected in CMV-plants (Figure 3A,B). The alteration of gene expression by transgene-expressed 2b protein was not positively correlation with the high proportion of Hypo-DMRs found in 2b-3 plants. Indeed, we previously found that most loci corresponding to Hypo-DMRs in 2b-3 plants were mapped to repetitive regions and TEs and that only a small number of loci were mapped to gene-coding sequences [31].

We then searched for genes that were induced and contained Hypo-DMRs in CMV- and 2b-3 plants. A total of 139 of 3363 (4.1%) and 208 of 1180 (17.6%) of induced genes in CMV- and 2b-3 plants were associated with Hypo-DMRs (Figure 3C). Only 19 genes contained Hypo-DMRs in their promoters (Figure 3C). These data suggest that CMV infection-induced gene expression was not always accompanied by 2b impairment of promoter DNA methylation. We proposed that downstream genes of signaling transduction pathways would be induced by upstream regulatory genes which might be induced due to 2b-mediated demethylation during CMV infection.

**Figure 4:**
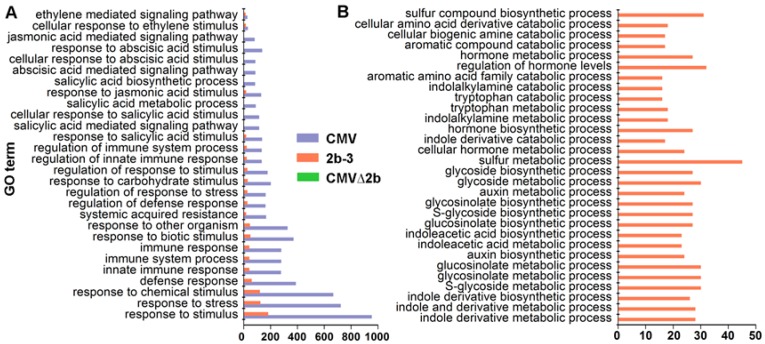
The specific enriched GO terms of genes induced in CMV-plants (**A**) and 2b-3 plants (**B**). Neither specific in plant immunity pathways nor metabolism pathways were found in CMV∆2b-plants.

Notably, GO analysis showed that CMV-infection specifically induced genes enriched in plant immunity pathways, and only a few of these genes were found to be induced in 2b-3 plants (Figure 4A). Taking into account the complexity of RNAi in plant development, it was sound that the majority of induced genes in 2b-3 plants were involved in extensive metabolism pathways (Figure 4B). Neither specific genes involved in plant immunity pathways nor specific genes involved in metabolism pathways were found in CMV∆2b-plants (Figure 4), which likely attributed to the weak pathogenicity of CMV∆2b lacking 2b VSR activity [32,34].

Taken together, the transcriptome sequencing analysis reveals that the 2b suppressor activity resulted in differential gene expression in the presence or absence of CMV infection. In the context of viral infection, the 2b-associated effect conspicuously triggers plant immunity pathways.

### 3.3. 2b-Dependent Triggered Plant Immunity in The Context of CMV Infection

**Figure 5:**
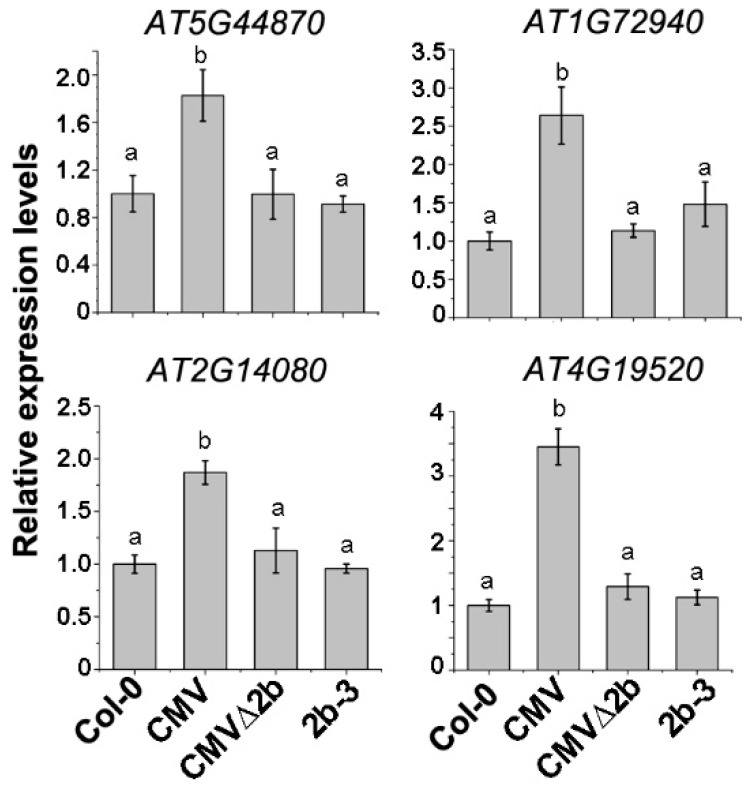
The relative expression of R genes in CMV-, CMVΔ2b- and 2b-3 plants. Means identified by different letters are significantly different from each other. Error bars represent SEs from three biological replicates. One-way ANOVA followed by Tukey’s multiple comparisons test, was used for statistical analysis (*P* < 0.05).

To evaluate the above transcriptome data in correlation with the 2b-associated Hypo-DMRs, four disease resistance protein (R) genes (*AT5G44870*, *AT1G72940*, *AT2G14080* and *AT4G19520*) in which Hypo-DMRs, especially in the CHH context, were detected in the promoters (Figure S2) and whose expression was induced in CMV-plants were selected for RT-qPCR. Consistent with the transcriptome data, the expression of these four genes was induced in CMV-plants (*P* < 0.05, one-way ANOVA) but not in CMVΔ2b- and 2b-3 plants (Figure 5), demonstrating that induced expression of these R genes occurred in only CMV infection and required the function of 2b. CMV∆2b infection in the absence of the 2b protein or transgene-expressed 2b protein would not trigger the expression of these *R* genes, despite the detection of CHH Hypo-DMRs in promoters of the four *R* genes in 2b-3 plants (Figure S2).

**Figure 6:**
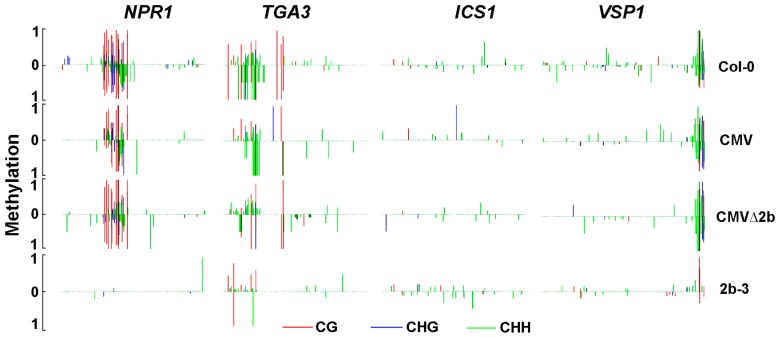
The methylation level in the promoters of genes involved in SA pathway. Vertical lines show the methylation level of each cytosine in Col-0, CMV-, CMVΔ2b- and 2b-3 plants.

**Figure 7:**
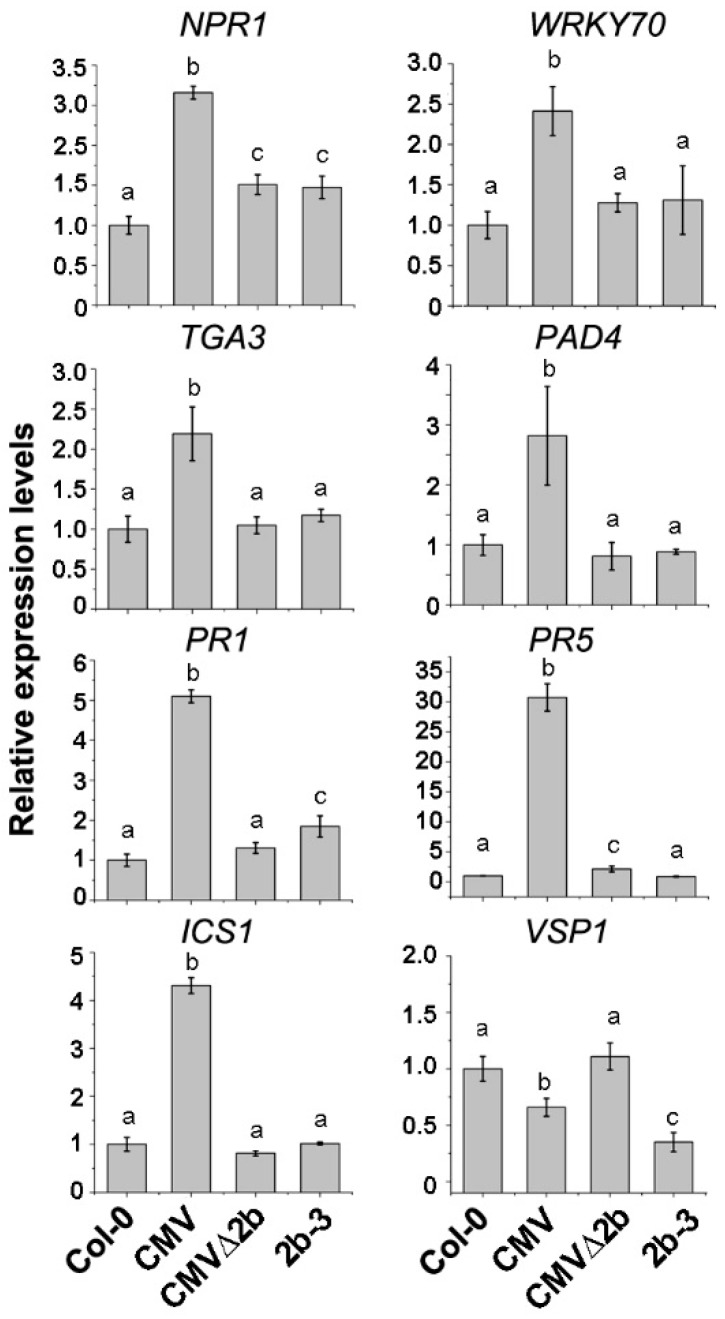
The relative expression of genes in SA and JA pathways in CMV-, CMVΔ2b- and 2b-3 plants. Means identified by different letters are significantly different from each other. Error bars represent SEs from three biological replicates. One-way ANOVA followed by Tukey’s multiple comparisons test was used for statistical analysis (*P* < 0.05).

The *NPR1* gene, which was required to establish systemic acquired resistance through SA signaling, was also selected for RT-qPCR confirmation because its promoter contains Hypo-DMRs in both CMV- and 2b-3 plants (Figure 6). Consistent with the transcriptome data, compared to wild-type Col-0, the expression of *NPR1* was significantly increased in CMV-plants (*P* < 0.05, one-way ANOVA) (Figure 7). The induction of *NPR1* in 2b-3 plants and CMV∆2b-plants was much lower than that in CMV-plants (*P* < 0.05, one-way ANOVA) (Figure 7). These data again demonstrate that CMV∆2b infection and transgene-expressed 2b protein would not trigger strong plant immunity, even though 2b-mediated strong demethylation in the *NPR1* promoter (Figure 6). This result further supports the viewpoint that CMV-triggered plant immunity requires the effect of 2b-mediated demethylation on the *NPR1* promoter and induction of *NPR1* expression in the context of CMV infection.

In CMV-plants, transcriptome data on induced expression include other genes in the SA signaling pathway that are directly or positively regulated by *NPR1*, such as *WRKY70* and *TGA3* [36]. RT-qPCR analysis also confirmed the induction of both *WRKY70* and *TGA3* genes (Figure 7). Induced expression of these genes was not detected in either CMV∆2b- or 2b-3 plants (Figure 7), although reduced methylation of the *TGA3* promoter was observed in both CMV∆2b- and 2b-3 plants (Figure 6), suggesting that induction of *TGA3* did not depend on changes in methylation, consistent with the result that *TGA3* is positively regulated by *NPR1* [36]. Similarly, in CMV-plants, transcriptome data on the induction of *PAD4*, *PR1* and *PR5*, which have been detected in response to SA-treatment [36], were confirmed by RT-qPCR (Figure 7). Consistent with the transcriptome data, the induction of *PR1* and *PR5* in CMV∆2b- or 2b-3 plants was much lower than that in CMV-plants (Figure 7).

In addition to downstream components in the SA pathway, *ICS1*, a component of the major route for SA biosynthesis in immunity [19], was also significantly induced in CMV-plants, but not in CMV∆2b- or 2b-3 plants, and this result was confirmed by RT-qPCR (Figure 7). The fact that a low methylation level and no Hypo-DMRs were found in the *ICS1* promoter in either genotypic plant (Figure 6)suggested that the 2b-dependent increased expression of *ICS1* in CMV-plants did not result from the direct effect of 2b-mediated demethylation on the *ICS1* gene, presumably by enhancing certain transcription factor(s) or repressing inhibitor(s) in a manner dependent on or independent of the VSR activity of 2b, as shown for the 2b-JAZ interaction, which interferes with the JA pathway independent of VSR activity [38]. Indeed, we detected Hypo-DMRs in the promoter of *VSP1*, a JA-inducible gene, in 2b-3 plants (Figure 6); however, the expression of *VSP1* was reduced in CMV- and 2b-3 plants (Figure 7), suggesting that VSR-activity independent of 2b protein was involved in the alteration of *VSP1* expression.

Taken together, our data demonstrate that CMV induced defensive genes and that SA biosynthesis and signaling depended on the 2b protein. Evidently, the induction of only a few transcription factor or upstream regulator genes, directly resulted from the 2b-mediated demethylation in the context of CMV infection.

### 3.4. 2b-sRNA, But Not 2b-AGO, Binding Was Required for Triggering Plant Immunity during CMV Infection

**Figure 8:**
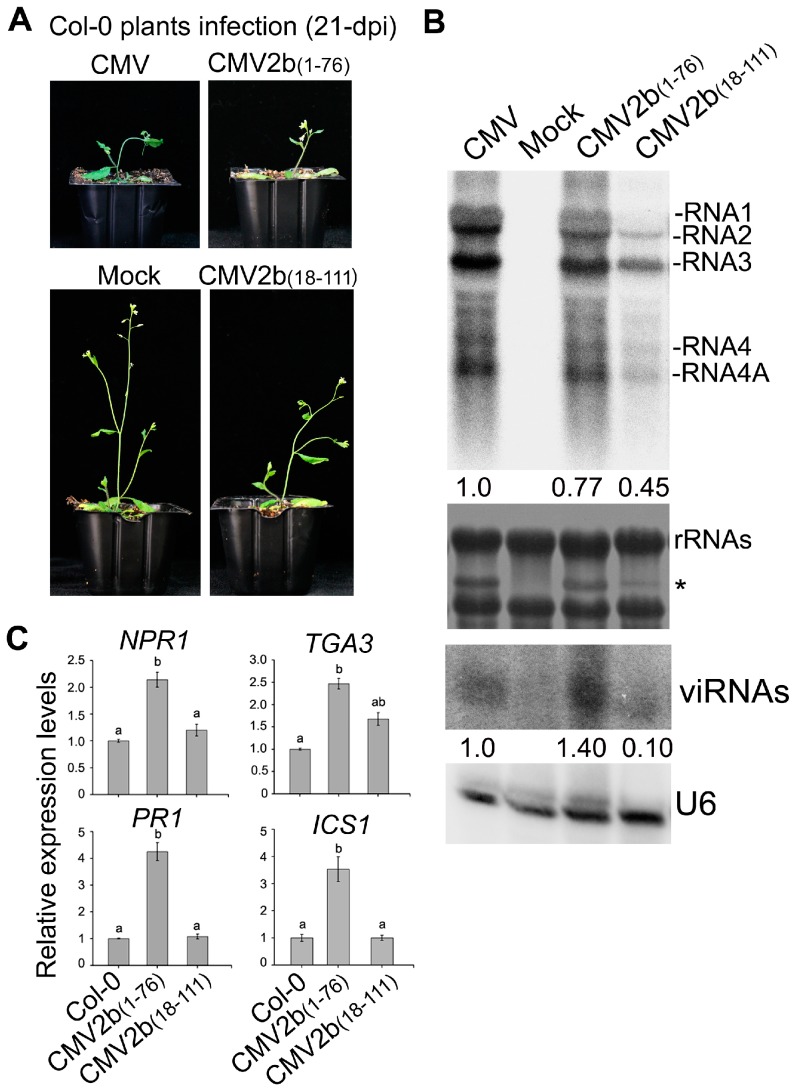
Pathogenicity and the relative expression of genes in SA pathways in CMV2b_(1–76)_- and CMV2b_(18–111)_-infected plants. (**A**) Disease symptoms and (**B**) viral RNAs and sRNA accumulation (21 dpi). * a stained viral RNA used as an indicator of CMV infection. viRNAs represented virus-derived siRNAs. The values below referred to the relative hybridization signal intensity of RNA 3 and viRNAs measured for each sample with the accumulation level in WT plants set as 1. (**C**) Virus-induced expression of defensive genes. Means identified by different letters are significantly different from each other. Error bars represent SEs from three biological replicates. One-way ANOVA followed by Tukey’s multiple comparisons test, was used for statistical analysis (*P* < 0.05).

Finally, we investigated how 2b-sRNA- and 2b-AGO- binding activities contribute to triggering plant immunity during CMV infection. We inoculated Col-0 with the CMV mutants CMV2b_(1–76)_ and CMV2b_(18–111)_, which express the 2b functional domains of sRNA or AGO binding, respectively, and tested the effect of these CMV mutants on the induction of defensive genes. The truncated 2b proteins stably expressed in the CMV2b_(1–76)_- and CMV2b_(18–111)_-infected plants respectively [32]. Similar to integral 2b protein, 2b_(1–76)_- and 2b_(18–111)_ were mainly detected in the nucleus especially in the nucleoli [30,32]. Consistent with our previous finding [32], CMV2b_(1–76)_ infection exhibited the same virulence as wild-type CMV infection but remarkably reduced viral RNA levels and increased viRNAs, whereas CMV2b_(18–111)_ infection reduced virulence and viral RNA levels (Figure 8A,B). The expression levels of *NPR1*, *PR1*, *TGA3* and *ICS1* were significantly induced in CMV2b_(1–76)_ infected-plants compared to wild-type Col-0 (*P* < 0.05, one-way ANOVA) (Figure 8C). Increased expression was not detected in CMV2b_(18–111)_-infected plants for most tested genes, although *TGA3* was slightly increased in CMV2b_(18–111)_-infected plants (*P* > 0.05, one-way ANOVA) (Figure 8). Taken together, our data demonstrate that 2b-sRNA-binding activity, which is responsible for virulence, triggers systemic acquired resistance through SA signaling, while 2b-AGO-binding activity likely does not play a role in triggering SA signaling but is required for countering silencing-mediated degradation of viral RNAs, a process that is RDR dependent [32].

## 4. Discussion

The 2b protein encoded by CMV exhibited complex activities, including the suppression of PTGS and TGS. Previous studies revealed that the 2b protein plays a role in the suppression of RdDM requiring sRNAs-binding activity but independent of the interaction with the AGO protein [31,45]. During CMV infection, 2b-sRNA binding activity is required for virulence, while the 2b-AGO interaction was found to be required for the suppression of RDR-dependent antiviral silencing during CMV infection [32]. In the present study, we further found that 2b-sRNA binding activity is required for the induction of genes enriched in plant immunity pathways during CMV infection, while the majority of induced genes in 2b-transgenic plants were involved in extensive metabolism pathways. We also show that 2b-dependent changes in DNA methylation were detected on a larger scale in 2b-transgenic plants than in CMV-infected plants. Our data clearly reveal the differences in 2b-dependent changes in DNA methylation and gene expression between CMV-infected and 2b-transgenic plants. When 2b was expressed by a transgene, in the absence of viral replication and virus-derived siRNAs accumulation, the nuclear localization and siRNA-binding capacity of the 2b protein would possibly bind a large amount of endogenous siRNAs and affect methylation in a wide range of DNA sequences [30]. However, when 2b was virally expressed during CMV infection, regardless of the amount of the 2b protein, a large amount of viRNAs were inevitably bound by the 2b protein, which would impair 2b protein binding to endogenous siRNAs and affect the impact of 2b on DNA methylation [45,46]. Moreover, the recent finding of a novel self-attenuation mechanism, in which the CMV CP protein inhibited the 2b protein and antagonized the suppression of host RNAi by 2b [37], might also account for the weak VSR effect of the 2b protein on endogenous genes during CMV infection. Nevertheless, consistent with the fact that VSRs have dual roles in maintaining the balance between plant development and virus accumulation [4], in addition to counteracting host antiviral silencing during CMV infection, the 2b protein can also alter host genomic DNA methylation and endogenous defensive gene expression.

Transcriptome and RT-qPCR analyses revealed that CMV infection induced the expression of a number of *R* genes (Figure 5), as well as SA biosynthesis and signaling (Figure 7), which required the presence of the 2b protein, consistent with recent reports that CMV infection induced host factors with antiviral effects and these effects relied on the function of 2b [47,48]. Although only a few of these genes were found to be related to the direct effect of 2b-mediated demethylation on their induction (Figure 3C), a large amount of 2b-dependent Hypo-DMR loci detected in CMV infection would also imply a defensive effect (Figure 4). Indeed, we previously found that many DMRs that mapped to repetitive sequences in 2b-3 plants [31] overlapped with those detected in CMV-plants. We also found that CMV infection in *Nicotiana benthamiana* resulted in the accumulation of a large amount of repetitive-derived siRNAs that were the origin for CMV satRNAs [33], which attenuated CMV disease symptoms and produced satRNA-derived siRNAs that target CMV RNAs for degradation [10,34]. Therefore, the effect of 2b-dependent demethylation on the induction of the host defense response does not simply reflect the induced expression of a few of endogenous defense genes.

It has been reported that SA accumulation was increased in Fny-CMV infected plants but not in Fny-CMV∆2b infected plants [36]. Consistently, we found that *ICS1*, which is essential for SA biosynthesis, was induced in a 2b-dependent manner during CMV infection but not in CMV∆2b- and 2b-3 plants (Figure 7). It is likely that accelerating SA accumulation during CMV infection required the function of 2b possibly via cooperation with other viral components. In addition to accelerating SA synthesis, the VSR activity of the 2b protein was required for host SA signaling during CMV infection. We detected that CMV induced significant expression of *NPR1* that depended on 2b-mediated demethylation of the *NPR1* promoter (Figure 6 and Figure 7). Consequently, a number of downstream components of the SA pathway, such as *WRKY70*, *TGA3*, *PR1* and *PR5*, were also significantly induced in CMV-plants, but not in CMV∆2b- and 2b-3 plants (Figure 7). Recent study has reported that a calmodulin-like protein (rgs-CaM) in tobacco functioned as an immune receptor for 2b protein and induced salicylic acid signaling in the presence of CMV virus infection or calcium signaling [49]. Also, *PR1* was not induced in 2b-expressed tobacco plants but it was induced under the calcium ionophore treatment [49]. These findings are also consistent with the previous finding that 2b expression increased the influence of SA on the transcriptome in comparison with the application of SA on Col-0 and Fny-CMV 2b transgenic plants [36]. Furthermore, infection with CMV mutants that expressed the 2b functional domains of sRNA or AGO binding, we found that CMV-induced SA signaling depends on 2b-sRNA binding activity. rgs-CaM exclusively recognized the sRNA-binding domain [50], which also indicated that the sRNA binding activity of 2b was required for induction of the immune response. 2b-mediated activation of SA signaling may lead to suppression of JA signaling, which benefits the transmission of CMV by its aphid vectors [36]. A recent study also showed that the 2b protein inhibited JA signaling by directly interacting with and repressing JA-induced degradation of JAZ proteins instead of using its VSR activity [38]. We also showed that inhibition of the JA-inducible gene *VSP1* depended on the 2b protein but was not mediated by demethylation (Figure 6 and Figure 7). Our GO analysis also showed that CMV-infection largely affected JA and other plant defense pathways (Figure 4). We propose that 2b-dependent alteration of DNA methylation also has a direct or indirect effect on these defense signaling pathways during CMV infection and is mediated by its VSR activity and/or by cooperation with other viral components, as well as by other yet to be identified functions.

In summary, by comparing bisulfite sequencing and transcriptome sequencing, we found that the VSR 2b protein had different effects on the induced expression of endogenous genes when it was constitutively expressed by a transgene or was expressed during natural viral infection. While the majority of induced genes in 2b transgenic plants were involved in extensive metabolism pathways, CMV infection specifically induced genes enriched in plant immunity pathways and was dependent on 2b-sRNA binding activity, which is also responsible for virulence. Our finding reveals that in addition to the suppression of host antiviral silencing that requires 2b-AGO interaction, the 2b VSR function is required for CMV-triggered host defense responses, especially SA signaling, in the context of viral infection. Therefore, the VSR 2b protein, with multiple functions, plays a key role in maintaining the balance between plant development and virus accumulation.

## Supplementary Materials

The following are available online at www.mdpi.com/link, Figure S1. The accumulation of 2b protein in 2b-3 plants. Ponceau S Staining was used as loading contro, Figure S2. The methylation level in the promoters of R genes. Vertical lines show the methylation level of each cytosine in Col-0, CMV-, CMVΔ2b- and 2b-3 plants. Table S1. List of primers used in this study.
