# Supplementary material for: CMV2b-Dependent Regulation of Host Defense Pathways in the Context of Viral Infection
Source: Viruses. 2018 Nov 9;10(11):618. doi: 10.3390/v10110618 (PMC6265714; doi:10.3390/v10110618)
Supplement: Supplementary file 1 [file viruses-10-00618-s001.zip › viruses-342789-SI/Table S1.docx]

**Table S1. List of primers used in this study.**

| Primer name | Primer sequence (5’ to 3’) | Purpose |
| --- | --- | --- |
| *AT4G33380*_F | GCCAAAACAGAATTTCAAAGACA | RT-qPCR |
| *AT4G33380*_R | ATTTCTCCCATCAATCTGTCCAC | RT-qPCR |
| *ICS1*_F | GATAGTAGCTGGAAGTGACCCAT | RT-qPCR |
| *ICS1*_R | CCCATACAAACAAAACAATCAAA | RT-qPCR |
| *PAD4*_F | GCTTATCCTCCGATGAACCTCTA | RT-qPCR |
| *PAD4*_R | TTGAATGGCCGGTTATCACCACC | RT-qPCR |
| *PR1*_*F* | CCATACTAATGAAGTAATGATGTG | RT-qPCR |
| *PR1*_R | TTAAATAGATTCTCGTAATCTCAG | RT-qPCR |
| *PR5*_F | TCGTGTTCATCACAAGCGGCATT | RT-qPCR |
| *PR5*_R | GCTGTCGGGAAGCACCTGGAGTC | RT-qPCR |
| *NPR1*_F | GTGTAAAGATAGCACCTTTCAGA | RT-qPCR |
| *NPR1*_R | GTCAAGTCCTCACAGTTCATAAT | RT-qPCR |
| *TGA3*_F | AAAAGTTGTTGAGTCTGTGAATC | RT-qPCR |
| *TGA3*_R | TGTCTTGTCGTCAATATCTTACTC | RT-qPCR |
| *WRKY70*_F | ACTTGAGGACGCATTTTCTTGGA | RT-qPCR |
| *WRKY70*_R | ACCTTCTGGACTTGCTTTGTTGC | RT-qPCR |
| *VSP1*_F | TCGAAGTTGACGCAAGTGGTGT | RT-qPCR |
| *VSP1*_R | TCCAGGAGTATCCTCAACCAAAT | RT-qPCR |
| *AT5G44870_*F | AAGGACTCAATCTCAACCTACGC | RT-qPCR |
| *AT5G44870_*R | GGCTCTAAGACTGATCCAACTGC | RT-qPCR |
| *AT1G72940_*F | ATTGACCAGTTTGGCTAGTATCTC | RT-qPCR |
| *AT1G72940_*R | TAGGTTCTTCCCATTGCTTATTG | RT-qPCR |
| *AT2G14080_*F | ACTAGGTAAAGAAATTGTTCGTA | RT-qPCR |
| *AT2G14080_*R | ATATCCCTATAACACTTCTACCA | RT-qPCR |
| *AT4G19520*_F | AATGTTCTTTGAATTGGGCATCG | RT-qPCR |
| *AT4G19520*_R | CATGGATTATCATGGTCGCAGTC | RT-qPCR |
