# Supplementary figures and images for: CMV2b-Dependent Regulation of Host Defense Pathways in the Context of Viral Infection
Source: Viruses. 2018 Nov 9;10(11):618. doi: 10.3390/v10110618 (PMC6265714; doi:10.3390/v10110618)

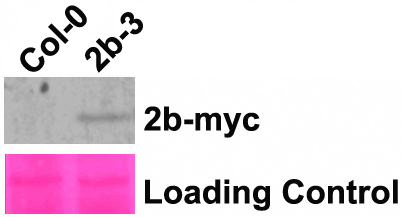

Supplement: Supplementary file 1 [file viruses-10-00618-s001.zip › viruses-342789-SI/Figure S1.jpg]

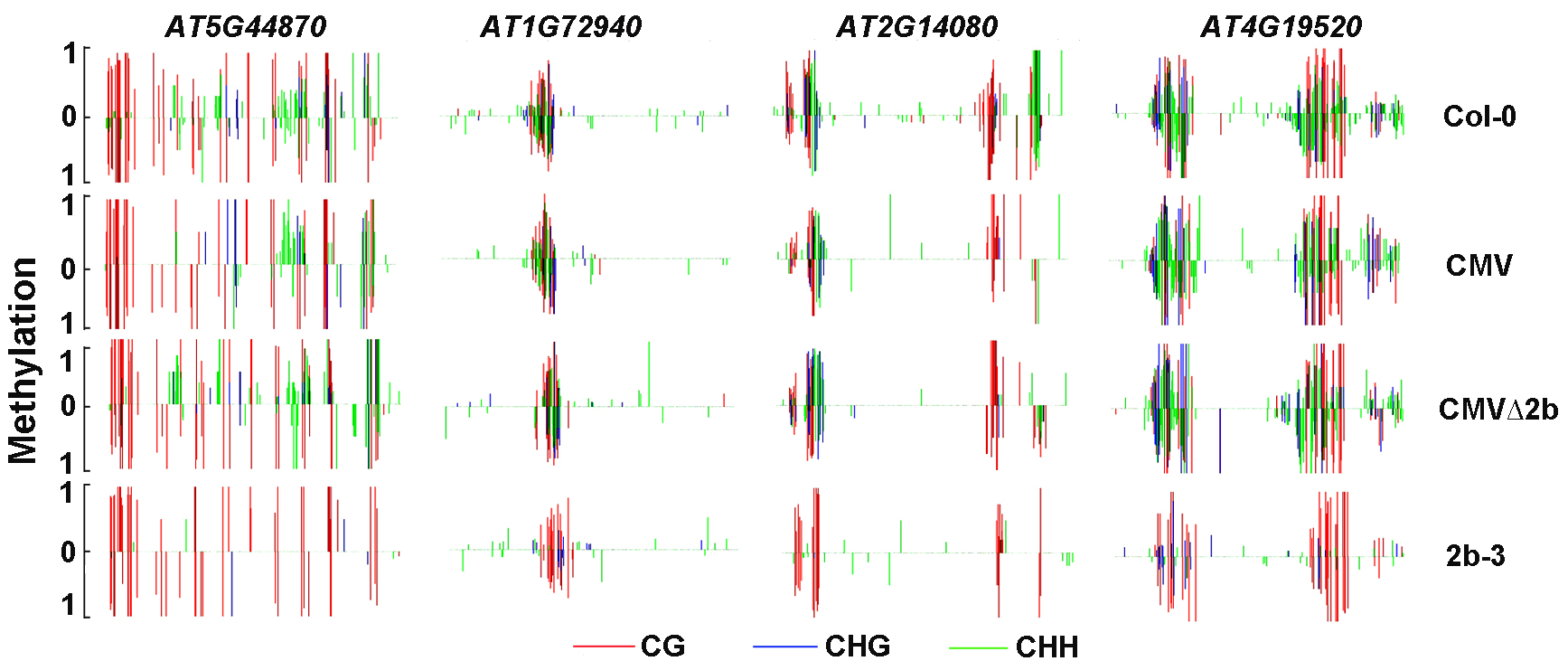

Supplement: Supplementary file 1 [file viruses-10-00618-s001.zip › viruses-342789-SI/Figure S2.jpg]
